# Impact of BASE skill training among women seeking outdoor services in Rajasthan, India

**DOI:** 10.6026/973206300212377

**Published:** 2025-08-31

**Authors:** Suresh Chand Bairwa, Deepika Kumari, Ratan Prakash Dhir, Mamta Mishra, Lokesh Parashar

**Affiliations:** 1 Department of Community Medicine, ESIC Medical College & Hospital, Alwar, Rajasthan, India; 2 Department of Anesthesiology, Government Medical College, Alwar, Rajasthan, India; 3 Department of Community Medicine, ESIC Medical College, Faridabad, Haryana, India; 4 Department of Microbiologist, Subharti Medical College, Meerut, Uttar Pradesh, India

**Keywords:** Breast self-examination, breast cancer, knowledge attitude & practices, quasi-interventional study

## Abstract

Breast Self-Examination (BSE) is an accepted screening method for early detection of breast cancer. Study aims to evaluate the effect of training of this skill in women's KAP. An opportunistic, quasi-interventional study was conducted May 2022 to December 2022 at the rural health training center (RHTC) area of S.M.S Medical College, Jaipur. A total of 330 women attending the outdoor event were trained in breast awareness & self-examination (BASE) skill and its impact in terms of breast cancer knowledge, attitude, and practice were followed up and assessed telephonically by a pre-designed, pre-validated questionnaire. It was found that 30.4% of the respondents showed an increase in knowledge, while 45.8% and 7% showed an increase in attitude and practice respectively. Thus, enhancement of breast cancer awareness is need. Further, focusing on recognized barriers by healthcare professionals with the involvement of spouses, family and community would have a substantial benefit in early detection of breast cancer.

## Background:

Breast cancer is a leading global health concern, with over 2.3 million new cases diagnosed each year. It ranks as the first or second most common cause of cancer-related deaths among women in 95% of countries worldwide [1]. In India, the incidence of breast cancer is alarmingly high, with 1.78 lakh women diagnosed in 2020, accounting for 13.5% of all cancer cases and 10.6% of cancer-related deaths (90,000 deaths) [2]. Early diagnosis of breast cancer is critical, as at least 60% of cases are diagnosed and treated in the early stages, offering better chances for successful treatment and recovery. Timely detection, particularly within 60 days of the initial presentation, significantly improves outcomes and survival rates [1]. Breast self-examination (BSE) has long been recognized as an important method for early breast cancer detection, especially in resource-limited settings where access to clinical screenings or mammography may be limited [3].

BSE empowers women to take an active role in monitoring their breast health and to detect potential abnormalities, such as lumps or changes in size, shape, or texture of the breast [4]. Studies have shown that BSE, along with increased breast awareness, can improve women's knowledge, attitudes and practices (KAP) related to breast health and early detection [5]. There is a need for improving women's understanding and ability to perform BSE, breast cancer can be detected early with better clinical outcomes, particularly in underserved regions [6, 7-8]. Therefore, it is of interest to evaluate the effectiveness of breast awareness and BSE skill training in enhancing women's KAP regarding BSE and early breast cancer detection.

## Methodology:

## Study design and setting:

This non-randomized, quasi-interventional study was conducted at Rural Health Training Centre (RHTC) in Nayla, Jaipur, under the Department of Preventive and Social Medicine (PSM), SMS Medical College, Jaipur between May 2022 and December 2022. The participants were women recruited by convenience sampling.

## IRB approval:

The study was conducted after approval of the Institutional Research Review Board of the Institute. This study was performed in lines with the Declaration of Helsinki.

## Consent to participate declaration:

Informed consent was taken from every eligible woman prior to participate in study.

## Study population:

All women ≥18 years of age and not been diagnosed with breast cancer, seeking outdoor services in RHTC Nayla and attached community health centre were enrolled in study. And women who were non-cooperative, lost to follow-up or not completing the questionnaire were excluded from the study.

## Data extraction and study variables:

## Data collection procedure:

After taking informed consent for participating in the study, pre-BASE questionnaires were employed. Based on the knowledge gaps found in the baseline data, breast awareness & self-examination (BASE) training program was created. A review of the literature, the WHO-recommended training manual, which included videos for breast self-examination (BSE) and other readily available educational resources were also considered. As a teaching tool, printed educational materials with a focus on BSE were employed. The researcher's educational intervention was a group-based instructional training session. The program for health education intervention comprises group discussions and demonstrations. The researcher discusses and demonstrates them in three hourly sessions. The training covers the overview of important topics related to breast self-examination, emphasizing its definition, common misconceptions, significance, application, technique and rules. Finally, it includes a practical demonstration of how, when, and what to look out for during BSE, along with group work aimed at enhancing BSE knowledge, attitude and practice. A video was included to provide a better picture of the step-by-step process of BSE performance. In addition, following each training session, the participants were free to ask questions. After BASE skill training sessions, impact of skill training in terms of breast cancer knowledge, attitude and practice were assessed on subsequent visit and follow-up telephonically. Follow-ups held for next four months. Trainings and follow-ups were done by female researchers for better response.

## Questionnaire:

A pre-designed, pre-validated questionnaire designed to assess knowledge, attitude, and practice (KAP) regarding BSE, included four dimensions: demographic characteristics, knowledge dimension (including knowledge of breast cancer and breast self-examination), attitude dimension, and practice dimension. The basic characteristics were covered by eleven items. The knowledge part included 15 items; correct answers were scored 1 point and wrong/unclear answers were scored 0 points, with a theoretical score range of 0-15 points. The attitude part consisted of thirteen items, using a 3-point Likert scale, from positive (3 points) to negative (1 point). The total score ranged from 13 to 39 points. The practice part included seven items, using a 3-point Likert scale, from positive (3 points) to negative (1 point). The total score ranged from 7 to 21 points. For relation between socio-demographic characteristics and pre-base knowledge scores, knowledge score divided in to two, ≥50% and <50% score. To find out the impact of BASE training scores were categorized into three levels: 'good' (>70%), 'average' (50-70%), and 'poor' (<50%). The questionnaires were distributed to the participants at Rural Health Training Centre (RHTC) in Nayla, Jaipur. Five doctors and nurses were responsible for promoting and distributing the questionnaires was trained for this study.

## Sample size:

Used to calculate the sample size where, "n" represents the sample size, "α" represents the type I error, which is typically set at 0.05, Zα/2 = 1.96, l represents the allowable error, typically set at 0.05 and "p" is prevalence of awareness of breast self-examination among women was 18% [9]. To account for attrition and lost to follow-up, the sample size was adjusted to 360 women for study purpose.

n=(Z α/2)^2^ P(1-P)/(l)^2^

## Statistical analysis:

SPSS 25.0 (IBM Corporation) was used for analysis. The pre-BASE and post-BASE questionnaires were gathered, cleaned, manually coded, and then input into the computer. Frequency tables, charts and mean knowledge, attitude and practices ratings were created using descriptive statistics. The continuous variables were presented as mean ± standard deviation (SD). Categorical variables were expressed as n (%). Chai-square test used to found association between socio-demographic characteristics of the study participants and their pre-base knowledge scores regarding breast self-examination. The Spearman analysis was used to analyze the correlation of knowledge, attitude and practice scores. To display the mean scores and standard deviations at the pre-BASE and post-BASE, paired sample t-test statistics were employed. P < 0.05 consider as significant value.

## Results:

Out of 360 women, 30 women did not respond or were lost to follow-up and 330 respondents were enrolled in the study among 330 women, 297 (90%) being married. The mean age of the participants was 28.5 years, with a standard deviation of 9.42 years. Figure 1 (see PDF) show a significant proportion 264 (80%) of the study participants belong to age group 18-30 years and 198 (60%) illiterate and 270 (82%) were homemakers by occupation. In terms of religion, 228 (69%) of the participants identified as Hindu and 139 (42%) belonged to the Scheduled Caste (SC) community. Figure 2 (see PDF) shows that most of the participants were in Class III as per modified BG Prasad Socio-economic Scale 2023. Chi-square tests were applied to assess the relationship between socio-demographic characteristics and the pre-Breast Self-Examination (BSE) knowledge level of the participants. Table 1 (see PDF) reveals that marital status, education, occupation, religion, caste and socio-economic status were significantly associated with knowledge of breast self-examination (P-value <0.05). Table 2 (see PDF) demonstrates that the knowledge, attitude and practice (KAP) scores were significantly correlated with each other (P-value <0.05), highlighting the interdependence of these factors in shaping breast health awareness. To assess the impact of Breast Awareness and Self-Examination (BASE) training, a paired T-test was conducted to compare the pre- and post-training KAP scores. Table 3 (see PDF) shows that the KAP scores towards BSE significantly increased after BASE training (P-value <0.001). Post-training, the proportion of respondents with a good score (>70%) for knowledge, attitude and practice were 30.4% (100 participants), 45.8% (151 participants) and 7.0% (23 participants), respectively.

## Discussion:

The findings of this study provide valuable insights into the impact of Breast Self-Examination (BSE) education through Breast Awareness and Self-Examination (BASE) training on women in a resource-limited setting. A total of 330 participants were included, with 90% (297) being married and a significant proportion of the women 60% (198) being illiterate. The study revealed a significant improvement in knowledge, attitude and practice (KAP) towards BSE following the BASE intervention. The socio-demographic factors such as marital status, education, occupation, religion, caste and socio-economic status (SES) were significantly associated with BSE knowledge, highlighting the importance of considering these factors when designing health interventions. Regard socio-demographic profile, majority of respondents were young (mean age 28.5 years), illiterate (60%), and homemakers (82%), with a significant representation from lower SES (Class III). These characteristics are consistent with study conducted by Gray *et al.* [10], which found that most participants in rural Indian settings were young, illiterate women from lower SES backgrounds. Similarly, Sawhney *et al.* [11] found that women with low levels of education and from economically disadvantaged backgrounds were less likely to engage in health-promoting behaviors like BSE. The association between socio-demographic factors and BSE knowledge observed in this study (P-value <0.05) mirrors the findings of Singh *et al.* [12], who reported that higher education and better SES were linked to greater awareness of BSE in their study on rural women in India. This emphasizes the importance of targeting educational interventions to those with limited access to formal education and from lower socio-economic groups, where the need for breast cancer awareness is often most critical. In context of Impact of BASE training, key finding was the significant increase in KAP scores towards BSE after BASE training (P-value <0.001). The proportions of respondents achieving good scores (above 70%) for knowledge, attitude, and practice were 30.4%, 45.8%, and 7.0%, respectively. This indicates that while knowledge and attitude improved substantially, the translation of these improvements into actual practice was more limited. This finding is consistent with studies by Nisha *et al.* [13] who reported significant improvements in knowledge and attitudes about breast cancer and BSE among rural women following an educational intervention. However, the proportion of women practicing BSE regularly remained relatively low, similar to our study's findings. For instance, Kumarasamy *et al.* [14] found that while educational interventions significantly improved knowledge and attitudes about BSE, only a small fraction of women (12%) were practicing BSE regularly, despite understanding its importance, this disparity between knowledge and practice is a common challenge in health education interventions and may be influenced by factors such as fear, stigma, or lack of privacy, which prevent women from performing BSE consistently. Sawhney *et al.* [11] also noted that even when women were knowledgeable about BSE, socio-cultural factors, particularly in rural areas, made regular practice difficult.

The study found a strong positive correlation between knowledge, attitude and practice scores (P-value <0.001), which is consistent with other studies. Gray *et al.* [10] also reported a significant positive relationship between these three components in rural women. This correlation suggests that increasing awareness and improving attitudes towards BSE can positively influence self-reported practices. However, as seen in our study, while the correlation is significant, it is not always reflected in actual behavior change. This highlights the complexity of translating knowledge into consistent practice, which can be influenced by psychological, cultural, and environmental factors. One possible explanation for this observed gap is the "knowledge-action gap" in health behavior research, which suggests that while individuals may be aware of the health benefits of certain behaviors (such as BSE), other factors, including perceived barriers, lack of support, or cultural norms, prevent them from engaging in these behaviors regularly. This finding was echoed by Sawhney *et al.* [11], who explored the barriers to BSE practice in rural India and identified factors such as fear of detection, stigma and lack of privacy as significant deterrents. Although the BASE training led to significant improvements in knowledge and attitude, only a small percentage of participants 23 (7%) reported good practice scores post-intervention. This low proportion of regular BSE practice suggests that while educational programs can increase awareness, they may not be sufficient to overcome the socio-cultural and psychological barriers that prevent women from regularly performing BSE. Studies like Kumarasamy *et al.* [14] have shown that barriers such as fear of detecting abnormalities, embarrassment, lack of privacy and the perceived irrelevance of BSE in low-risk women can all inhibit the practice of BSE, even when knowledge and attitudes are positive. The role of social support in overcoming these barriers cannot be overstated. Wagle *et al.* [15] found that women who received support from family members or healthcare providers were more likely to engage in regular BSE. Future interventions may need to focus not only on knowledge dissemination but also on creating an enabling environment that supports women in overcoming these barriers, such as providing privacy, emotional support, and reassurance.

## Limitations and future research directions:

This study is not without limitations. Firstly, the study relied on self-reported data to assess BSE practices, which may be subject to social desirability bias. Future studies could incorporate objective measures, such as clinical breast exams or provider-reported assessments of BSE practice, to provide a more accurate measure of behavior change. Additionally, this study was cross-sectional in nature, meaning that it cannot determine the long-term effects of BASE training on sustained BSE practice. To address these limitations, future research could focus on longitudinal studies to track the impact of BASE training over time, as well as comparative studies assessing the efficacy of different educational interventions in improving both knowledge and actual practice of BSE. Moreover, examining the psychological impact of such programs-particularly how they influence anxiety and perceived risk-would provide a more comprehensive understanding of the outcomes of BSE education.

## Conclusion:

BASE training effectively enhances knowledge and attitudes about breast self-examination (BSE). However, it has limited impact on actual BSE practice. Key barriers include lack of proper technique, limited awareness of cancer signs and insufficient support from family and peers. Future programs should address socio-cultural and psychological obstacles, involve community and family support and provide on-going education and to skill-based training, especially in resource-limited settings.

## Authors' contribution:

Bairwa SC and Kumari D contributed to the concept and design of the study with data collection and supervised the project. Parashar L was the PhD Research Scholar responsible for doing the statistical analysis. Dhir RP drafted the manuscript and prepared the graphs and tables. All authors approved the final version of the manuscript.

## Informed consent:

Informed consent was taken from all before participating in study.

## IRB approval:

The study was conducted after approval of the Institutional Research Review Board of S.M.S Medical College, Jaipur. This study was performed in lines with the Declaration of Helsinki.

## Funding:

No funding was received for conducting this study.

## Database Availability Statement:

Authors have datasheet available in Microsoft Excel, will be share if requested.
